# Mediator Microbial Biosensor Analyzers for Rapid Determination of Surface Water Toxicity

**DOI:** 10.3390/s22218522

**Published:** 2022-11-05

**Authors:** Anna Kharkova, Vyacheslav Arlyapov, Anastasia Medvedeva, Roman Lepikash, Pavel Melnikov, Anatoly Reshetilov

**Affiliations:** 1 Department of Chemistry, Tula State University, 92 Lenin Avenue, Tula 300012, Russia; 2 M.V. Lomonosov Institute of Fine Chemical Technologies, MIREA—Russian Technological University, Prospect Vernadskogo 86, Moscow 119571, Russia; 3 G.K. Skryabin Institute of Biochemistry and Physiology of Microorganisms, Federal Research Center “Pushchino Scientific Center for Biological Research of the Russian Academy of Sciences” (FRC PSCBR), Russian Academy of Sciences, 5 Nauki Avenue, Moscow 142290, Russia

**Keywords:** mediator biosensor, water toxicity, early-warning instrument, electroactive biofilms, electron transport mediator, bioelectrocatalysis

## Abstract

Microbial mediator biosensors for surface water toxicity determination make it possible to carry out an early assessment of the environmental object’s quality without time-consuming standard procedures based on standard test-organisms, and provide broad opportunities for receptor element modifying depending on the required operational parameters analyzer. Four microorganisms with broad substrate specificity and nine electron acceptors were used to form a receptor system for toxicity assessment. Ferrocene was the most effective mediator according to its high rate constant of interaction with the microorganisms (0.33 ± 0.01 dm^3^/(g × s) for yeast *Saccharomyces cerevisiae*). Biosensors were tested on samples containing four heavy metal ions (Cu^2+^, Zn^2+^, Pb^2+^, Cd^2+^), two phenols (phenol and p-nitrophenol), and three natural water samples. The «ferrocene- *Escherichia coli*» and «ferrocene-*Paracoccus yeei*, *E. coli* association» systems showed good operational stability with a relative standard deviation of 6.9 and 7.3% (14 measurements) and a reproducibility of 7 and 5.2% using copper (II) ions as a reference toxicant. Biosensor analysis with these systems was shown to highly correlate with the results of the standard method using *Chlorella algae* as a test object. Developed biosensors allow for a valuation of the polluted natural water’s impact on the ecosystem via an assessment of the influence on bacteria and yeast in the receptor system. The systems could be used in toxicological monitoring of natural waters.

## 1. Introduction

Many chemical compounds present in water can have a negative impact on the natural ecosystem. The main polluting components of aquatic environments are solutions of heavy metals and organic compounds. Water toxicity early warning is performed by biotesting and physicochemical methods of analysis.

Physicochemical methods, such as high-performance liquid chromatography [1] and atomic absorption spectroscopy [2], make it possible to accurately define the types of chemical toxicants and their concentrations, but do not determine the degree of the toxic effect of substances on the natural ecosystem and do not reveal synergistic and antagonistic effects. The toxic impact of sample components can be established only using biological techniques, which imply the use of various trophic levels of living organisms: fish [3,4], plants [5,6], invertebrates [7,8], and microorganisms [9,10,11]. Almost all biological methods, however, have inevitable drawbacks that hinder their application. For instance, techniques using algae, daphnia, and fishes are highly sensitive to toxic substances but are time-consuming and low-reproducible. As a rule, the testing times of methods based on algae, daphnia, and fish are 72, 24/48 and 48 h, respectively, which limits the possibility of early warning water toxicity. 

Biosensor express analysis is possible due to the application of microorganisms that have physiological reactions with pollutants similar to higher organisms. Compared with relatively high-level organisms, such as fish and daphnia, microorganisms are easily cultured and enable observation of the reaction to toxic substances over short times. Due to these advantages, it has become a tendency over the past 20 years to develop microbial methods for assessing the general toxicity of water [12]. The most successful and widely used technique is based on bioluminescent bacteria. Owing to the ease of operation, high sensitivity to many types of toxic substances, and short testing times (~1 h), bioluminescent bacteria have been commercialized and included in the list of standard ISO methods [13].

Disadvantages of bioluminescent biosensors are that the analytical signal—a change in the intensity of fluorescence—is significantly influenced by the turbidity of the test sample; however, the problem is solved when designing a biosensor [14]. A more significant difficulty in the development of devices for the rapid monitoring of toxicity is that microorganisms are significantly inferior in terms of sensitivity to standard test systems (daphnia, algae, infusoria) [15,16]. One of the approaches to the development of a sensitive express system is the use of a stable microbial association that is sensitive to various kinds of toxicants (heavy metals, organic substances). With the luminescent type of toxicity detection, it is quite difficult to vary the biomaterial, so special attention is paid to the electrochemical biosensors [17,18,19,20,21,22,23,24,25,26,27,28,29]. Unlike luminescent biosensors, the amperometric type of detection applies to a significant number of single microorganisms and forms of bioreceptors based on consortia. Quite often, the main biomaterials used to assess toxicity are *E. coli* bacteria [19,23,24], *S. cerevisiae* yeast [19,25], and activated sludge [21,26]. Thus, the possibility of creating a whole-cell electrochemical biosensor based on the mediator p-benzoquinone for detecting heavy metal ions present in wastewater was investigated [17]; the biomaterial included an association of microbial strains such as *E. coli*, *B. subtilis*, and *S. cerevisiae*, which allowed for increasing the sensitivity of registration compared with the use of single *E. coli* microorganisms and the mediator [28]. The results have shown that an electrochemical biosensor based on a consortium of microorganisms and the mediator p-benzoquinone has a high sensitivity in assessing the toxicity of wastewater. 

Another approach to increasing sensitivity is to refine electron transfer processes. Electron transfer can be improved by nanomaterials. Thus, in [29], it is suggested to use *E. coli* bacteria immobilized in a chitosan polymer to assess the toxicity of aqueous media. Thionine adsorbed on the surface of the biomaterial is used for electron transfer from *E. coli* to the electrode. To increase the sensitivity of the system, a glassy carbon electrode is modified by carbon nanodots. The sensor was tested on 18 samples of natural water from a river, reservoir, and lake and showed a good correlation with the results of the standard luminescent method (R^2^ = 0.9827). The disadvantage of this technique of forming the receptor system is the possible leaching of the mediator from the analytical system, and consequently, the loss of stability. Another approach to refine electron transfer was used in our previous study [27], where choosing an electron transport mediator for *P. yeei* bacteria was based on a kinetic study. The most effective system was a biosensor based on the mediator ferrocene, since this redox-active compound has the highest rate of interaction with the biomaterial used—*P. yeei* bacteria—and the highest rate of electron transfer to the electrode. This approach made it possible to create a laboratory model of a biosensor for determining the toxicity of model wastewater using microorganisms with high sensitivity to toxic substances. Despite the successful use of the kinetic approach for choosing an electron transport mediator (the developed biosensor was more sensitive to heavy metals than luminescent bacteria), the express system was inferior in sensitivity to the standard test object, duckweed. This paper discusses the application of this approach with other microorganisms, which will allow for further formation of sensitivity associations to be able to reach the sensitivity of standard test objects.

The goal of the study is the creation of a more sensitive system for assessing natural water toxicity using the association of microorganisms that are sensitive to various classes of toxicants; increasing the efficiency of electron transfer by selecting a more effective acceptor based on kinetic research. For creating a more sensitive system, it is necessary to ensure the selectivity of toxicity analysis with respect to other integral water quality parameters; for example, biochemical oxygen demand (BOD). The novelty of this research is increasing the selectivity of the analysis by changing the method of the analytical signal recording, which makes it possible to measure the toxicity of those toxicants that are oxidized by the biomaterial. Thus, the approach proposed in this study differs between the toxicity index and biochemical oxygen demand (BOD).

## 2. Materials and Methods

### 2.1. Reagents and Materials

Dialysis membrane (Roth, Germany) with a 14 kDa cutoff, graphite powder (Fluka, Germany), and mineral oil (Helicon, Moscow, Russia) were used to form the working graphite paste electrode. All media for the cultivation of microorganisms were sterilized in a VP-01/75 autoclave (TZMOI, Moscow, Russia) at a pressure of 1 atm and at a temperature of 120 °C. As electron transport mediators, use was made of ferrocene (Aldrich, Darmstadt, Germany), 1,1′dimethylferrocene (Aldrich, Darmstadt, Germany), ferrocenecarboxaldehyde (Aldrich, Germany), ferroceneacetonitrile (Aldrich, Darmstadt, Germany); neutral red, 2,6-dichlorophenolindophenol, thionine, methylene blue, and potassium hexacyanoferrate(III) (purchased in Diaem, Moscow, Russia). All salts used to grow Chlorella vulgaris (Tamiya medium) [30], KH_2_PO_4_, MgSO_4_·7H_2_O, KNO_3_, FeC_6_H_5_O_7_, H_3_BO_3_, MnCl_2_·4H_2_O, ZnSO_4_·5H_2_O, MoO_3_, NH_4_VO_3_, Co(NO_3_)_2_·6H_2_O, were of CP grade (Diaem, Moscow, Russia).

### 2.2. Microorganisms

Bacteria *Pseudomonas veronii* DSM 11331T (16S rRNA gene sequence similarity, 99.79%) and *Paracoccus yeei* VKM B-3302 were isolated from activated sludge [30]. Yeast *Saccharomyces cerevisiae* VKM Y-1173 was kindly presented by the All-Russian Collection of Microorganisms, G.K. Skryabin Institute of Biochemistry and Physiology of Microorganisms, FRC PSCBR, Russian Academy of Sciences (IBPhM RAS); bacteria *Escherichia coli* K802 was provided by the Laboratory of Biosensors, IBPhM RAS.

### 2.3. Cultivation of Microbial Cells

Cells of strains *P. yeei*, *Ps. Veronii*, and *E. coli* were grown on an LB medium. The liquid medium had the following composition: tryptone, 10 g/dm^3^; yeast extract, 5 g/dm^3^; sodium chloride, 10 g/dm^3^. The cell growth medium was sterilized by autoclaving at a pressure of 1.15 atm for 45 min. Cells were grown aerobically for 20–24 h in 750-cm^3^ shake flasks at a temperature of 29 °C. The biomass obtained was centrifuged at room temperature at 10,000 rpm for 10 min and washed twice with a 20-mM phosphate buffer, pH 6.8, to remove the culture medium. The sedimented cells were resuspended in fresh buffer, distributed by portions and sedimented on an Eppendorf centrifuge for 10 min at 10,000 rpm. The washed biomass was weighed and kept in microtubes at a temperature of +4 °C.

Yeast *S. cerevisiae* was grown on a rich mineral medium (liquid glucose-peptone nutritious medium). The following composition of the liquid medium was used: glucose, 6.25 g/dm^3^; peptone, 6.25 g/dm^3^; yeast extract, 3.75 g/dm^3^; K_2_HPO_4_, 0.35 g/dm^3^. The cell growth medium was sterilized by autoclaving at a pressure of 1.1 atm for 45 min. Cells were aerobically grown for 18–20 h in 750-cm^3^ shake flasks at a temperature of 29 °C. The biomass obtained was centrifuged at room temperature for 10 min (10,000 rpm). The centrifugate was washed twice with a 20-mM phosphate buffer, pH 6.8. The sedimented cells were transferred into a fresh buffer, distributed by portions and sedimented on an Eppendorf centrifuge for 10 min at 10,000 rpm. The washed biomass was weighed and kept in microtubes at a temperature of +4 °C.

### 2.4. Formation of Working Electrode

The working electrode was formed by modifying a graphite paste electrode with electroactive material. For this purpose, a 10-mg weight of the mediator was dissolved in 500 μL of acetone and added to 90 mg of graphite powder (the mass fraction of ferrocene was 10% of the total mass of the powder). After the electrode was formed, microorganisms were applied on its surface as a suspension with a titre of 330 mg/mL in the amount of 10 μL. Thus, the specific mass of microorganisms on the surface of the carbon paste electrode was 0.52 mg/mm^2^. A dialysis membrane was used to fix microorganisms on the electrode surface.

### 2.5. Registration of Current–Voltage Characteristics

Cycling voltammograms were registered in a three-electrode cell using an Ekotest-VA voltammetric analyzer (Ekoniks-Ekspert, Russia). A carbon paste electrode with immobilized cells served as a working electrode; a platinum electrode served as an auxiliary electrode. A saturated silver/silver chloride (Ag/AgCl) electrode, in reference to which all voltammograms were presented, was used as a reference electrode. Measurements were conducted at a potential scan rate of 10 up to 200 mV/s in a 0.15 M potassium sodium phosphate buffer (pH 6.8) at a temperature of 22 °C. The cell volume was 15 mL. The rate constants of the interaction of electron-transport mediators with microorganisms are given in Table 1.

### 2.6. Biosensor Measurements

To register the dependence of the current on time, we used an IPC Micro galvanopotentiostat (Volta, Russia). Measurements were carried out at a constant potential of 0.25 V. 

After establishing a stable current level, an aliquot of the analyzed glucose solution was injected into the cell with a micropipette. After that, an aliquot of a mixture of a toxicant solution with glucose was introduced into the washed cell. After each measurement, the cell was washed with a potassium sodium phosphate buffer solution. As a result of the action of the toxicant, the current was significantly reduced.

The inhibition index calculated by Formula (1) served as an analytical signal:

(1) $$I_{\mathrm{inh}}=\frac{\Delta I_{\mathrm{glucose}}-\Delta I_{\mathrm{glucose}+\mathrm{toxicant}}}{\Delta I_{\mathrm{glucose}}}100\%,$$

where Δ*I*_glucose_ is the current upon addition of the glucose solution; Δ*I*_glucose+toxicant_ is the current upon addition of the glucose solution with inhibitor. The toxicant inhibition indices are given in Table 2.

### 2.7. Reference Toxicity Tests: Assessment of Chlorella Growth Inhibition

Biotests using Chlorella were conducted in accordance with the normative documents [31]. A culture of the alga was grown on a 2% Tamiya medium; the culture had an optical density of 0.005. Under aseptic conditions, chlorella was inoculated into the controls and analyzed media. For 7 days of incubation, the experimental and control vessels were continuously illuminated with a fluorescent lamp. The experimental vessels were arranged in the incubator at random (temperature, 24 ± 2 °C). The growth characteristics of the algal culture were determined by turbidimetry on a photoelectrocolorimeter (wavelength, 525 nm; cuvette length, 1 cm).

The relative difference of the average value of optical density for each dilution, as compared with the control, was calculated by the following formula:

(2) $$I=\frac{A_{cp0}-A_{cp}}{A_{cp0}}\cdot 100\%$$

where *A*_*cp*0_ is the average optical density of a control experiment; *A_cp_* is the optical density of an analyzed sample. 

When the toxicity exceeded 20% (inhibition), we calculated the *TDF*, toxicity dilution factor (see Formula (3)). When the toxicity factor exceeded 30% (stimulation), the value of *TDF* was calculated by Formula (4). The quality of water was established by the largest value of dilution, for which the toxicity criterion was exceeded.

(3) $$TDF=10^{\frac{\left(\log P_{g}-\log P_{s}\right)\left(I_{s}-0.2\right)}{I_{s}-I_{g}}+\log P_{s}}$$

(4) $$TDF=10^{\frac{\left(\log P_{g}-\log P_{s}\right)\left(I_{s}-0.3\right)}{I_{s}-I_{g}}+\log P_{s}}$$

where *P*_g_ is the (greatest) dilution value at which the deviation index was lower than the toxicity criterion; *P*_s_ is the (smallest) dilution value at which the deviation index was higher than the toxicity criterion; *I*_g_ and *I*_s_ are the values of indices *I* (expressed in fractions) corresponding to these dilution indices.

### 2.8. Biosensor Determination of Water Sample Toxicity

The analyzed water samples for biosensor analysis were prepared similarly to samples for testing algal culture inhibition [31]. As a result, a series of solutions for each water sample were prepared: 100, 33, 11, 3.7, and 1.2% (a series of dilutions with a triple decrease was presented). To each analyzed solution, glucose at a concentration of 0.1 mol/dm^3^ was added. The degree of inhibition of a bioreceptor element’s metabolic activity (IC) served as a registered analytical signal. By the obtained data, the toxic–nontoxic transition between the two dilutions was determined and the *TDF* was calculated. The *TDF* in the case of inhibition or stimulation was calculated by Formulas (3) and (4).

## 3. Results

### 3.1. Choice of Analytical Signal Registration Method

The electrochemical registration of the analytical signal in microbial mediator biosensors for toxicity assessment is based on the decrease of the metabolic activity of microorganisms that provide the basis of the test system. The stationary current decreases, due to the impact of a toxicant on the metabolic activity of the microorganisms, if an analyzed sample is added into the measuring cuvette [17,22]. The resulting current that reflects the metabolic state of cells can be converted into equivalent percentage values of inhibition according to Expression (5):

(5) $$I_{\mathrm{inh}}=\frac{I_{1}-I_{2}}{I_{1}}\cdot 100\%,$$

where *I*_1_ is the steady-state current before the addition of the toxicant; *I*_2_ is the current in a steady state after the addition of the toxicant [17,22].

Easily oxidizable organic substances may be present in natural water. When conducting the toxicological monitoring of natural waters, it can be expected that the addition of a sample to the biomaterial–mediator–electrode analytical system will lead to an increase in the metabolic activity of the biomaterial, which will oxidize the organic substances of the sample. At the same time, the amount of the reduced form of the mediator will increase. However, the reverse oxidation of the reduced form of the mediator on the electrode will contribute to an increase in the current strength recorded by the amperometric converter –this is the basis for the principle of express detection of biochemical oxygen uptake by microbial mediator biosensors [30].

In order to create a bioreceptor system sensitive both to metals and organic substances, it is necessary to choose the most effective biomaterial. The following microorganisms were used for the study: bacteria *E. coli* K802, *P. yeei* VKM B-3302, *Ps. veronii* DSM 11331T, and yeast *S. cerevisiae* VKM Y-1173. These microorganisms are quite sensitive to heavy metals and organic compounds, as they live in natural reservoirs that are not contaminated with pollutants. *E. coli* are used as a test object in biotesting methods [32]; these cells react to the presence of heavy metals in solutions and are widely utilized in the formation of biosensors to determine the integral toxicity of aqueous media [7]. *P. yeei* have been previously shown to be successful in assaying the toxicity of perfumery and cosmetic product extracts [30], and the total content of biodegradable organic substances in water [27,33]. *Ps. veronii* have been isolated from activated sludge [27]; these microorganisms have been shown to be capable of decomposing various aromatic compounds [34] and adsorbing heavy metal ions from wastewater [35]. With this in mind, all these bacteria are used for cleaning soils and wastewater from various pollutants, as well as for monitoring the state of the environment. The yeast *S. cerevisiae* is one of the currently well-studied microorganisms [36]; it is used for the biosorption of heavy metals [37], as well as in the development of biosensors for assessing the content of copper ions [38], for the analysis of water body toxicity [18].

**Figure 1:**
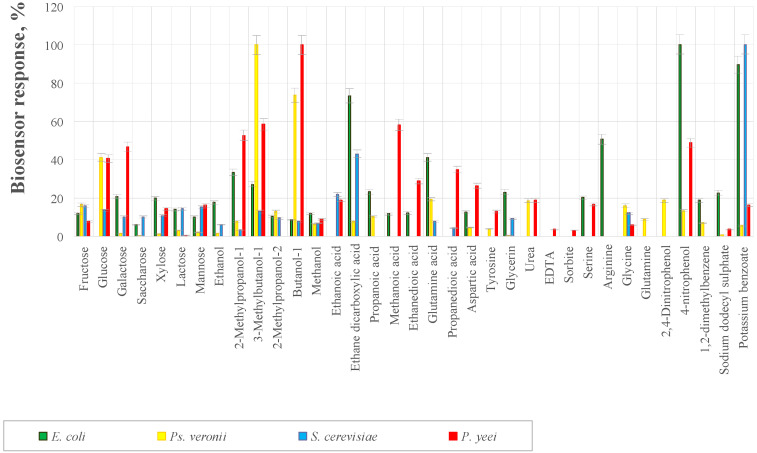
Substrate specificity of bioreceptor elements based on bacteria *E. coli* K-802, *Ps*. *veronii* DSM 11331T, *P. yeei* VKM B-3302, and yeast *S. cerevisiae* VKM Y-1173.

The character of the analytical signal under conditions of mediator bioelectrocatalysis will depend on the substrate specificity of the microorganism used in the receptor element relative to easily oxidizable organic compounds. The substrate specificity of the investigated receptor elements was assessed on 32 different substrates belonging to different classes of organic compounds (Figure 1). At the introduction of a substrate with a final concentration of 20 mM in the analyzed cuvette, the current strength increased. The difference between the final and initial values of the current strength was taken to be the biosensor response. The results on substrate specificity are presented as a percentage with respect to the maximum biosensor response.

**Figure 2:**
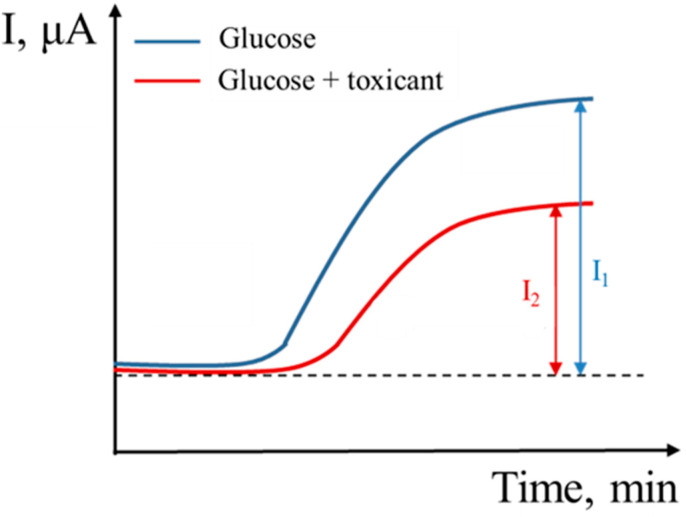
Scheme of analytical signal registration.

Microbial biosensors feature low selectivities; analytical responses were obtained for a significant number of different substrates that may occur in the reservoir (Figure 1). The narrowest substrate specificity is characterized by a receptor system based on *E. coli* bacteria, which is an advantage for determining toxicity. The low selectivity of the biosensor can become a problem in assessing the toxicity of natural waters. To minimize the influence of these substrates in determining toxicity, we chose another method of analytical signal registration (Figure 2) based on measuring the rate of glucose oxidation by microorganisms and the rate of glucose oxidation in the presence of a toxicant. 

Glucose was chosen as a model easily oxidizable substrate, which is metabolized by a wide range of microorganisms used for toxicity assessment. The degree of change in the current strength at the introduction of glucose solution in the presence and absence of a toxicant is an inhibition index that was used to assess toxicity. The use of this method of analytical signal registration considers the influence of easily oxidizable substrates occurring in reservoirs, and thereby reduces the error of the analysis.

### 3.2. Choice of Mediator and Microorganisms for Toxicity Index Assessment

When choosing an electron transport mediator, it is necessary to consider that the analytical signal is of a complex character, and is based on biochemical and electrochemical processes occurring in the analyte–microorganism–mediator–electrode system. Proceeding this, we comparatively assessed the effectiveness of bioelectrocatalytic oxidation of glucose by the investigated microorganisms and found the rate constants of mediator–biomaterial interaction.

**Figure 3:**
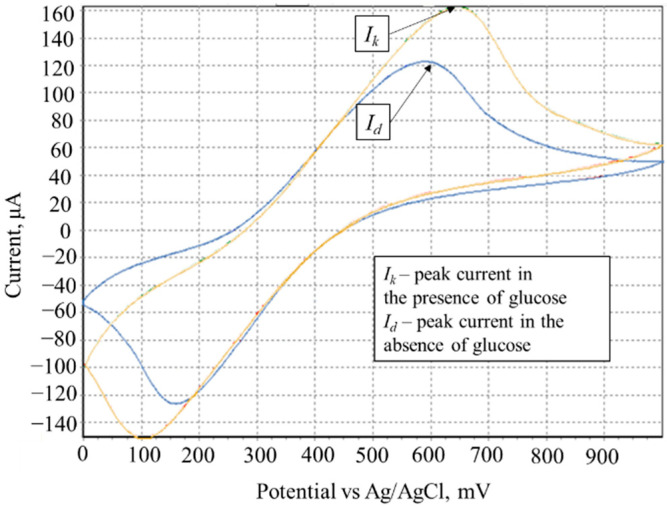
Determination of the constant of interaction of the mediator with *S. cerevisiae* yeast cells by cyclic voltammetry. The voltammograms based on ferrocene mediator (scan rate, 20 mV/s) in the presence of glucose (brown line) and in the absence of glucose (blue line) are presented.

At the registration of the cyclic voltammogram in the presence of glucose, there occurs an increase of anodic current (Figure 3) due to the electrocatalytic oxidation of substrate by the investigated biomaterial. To determine the rate constants of the interaction of mediator and biomaterial, we obtained the dependences of the ratio of the limiting anodic currents in the presence and absence of the substrate (*I_k_*/*I_d_*) on the value of the inverse scan rate 1/*ν*^1/2^; by the slope of the linear regression, the value of rate constant was found according to Expression (6).

(6) $$\frac{I_{k}}{I_{d}}=\sqrt{\frac{k\left[E\right]RT}{nF\nu }}$$

where *I_k_* is the limiting current in the presence of substrate (A); *I_d_* is the limiting current in the absence of substrate (A); *k* is the rate constant of mediator–biomaterial interaction, dm^3^/mg × s; *R* is the universal gas constant, J/mol × K; *T* is the temperature, degrees Kelvin; [*E*] is cell titre, mg/dm^3^; *ν* is scan rate, V/s; *n* is the number of transferred electrons; *F* is the Faraday constant, C/mol.

The obtained constants of the interaction of mediators with investigated microorganisms, as well as the rate constant of the heterogeneous transfer of electrons to the electrode, obtained earlier for the studied mediators [27], are given in Table 1.

**Table 1:** The rate constants of the interaction of electron-transport mediators with investigated microorganisms and the rate constant of the heterogeneous transfer of electrons to the electrode.

Mediator	Rate Constants of the Interaction of Mediators with Investigated Microorganisms, dm^3^/g × s				Rate Constant of the Heterogeneous Transfer to the Electrode, cm·s^−1^ [27]
	*Ps. Veronii*	*E. coli*	*S. cerevisiae*	*P. yeei* [27]	
Methylene blue	2.0 ± 0.5	0.20 ± 0.04	0.06 ± 0.01	0.021 ± 0.001	0.025 ± 0.009
Thionine	0.05 ± 0.01	0.010 ± 0.002	0.5 ± 0.1	0.013 ± 0.004	0.022 ± 0.005
Neutral red	0.020 ± 0.003	0.08 ± 0.01	0.6 ± 0.1	0.013 ± 0.003	0.017 ± 0.005
Potassium hexacyanoferrate(III)	0.20 ± 0.03	0.010 ± 0.002	0.002 ± 0.001	0.019 ± 0.003	0.0067 ± 0.0009
2,6-Dichloro-phenolindophenol	0.08 ± 0.02	0.010 ± 0.002	0.3 ± 0.1	0.013 ± 0.002	0.069 ± 0.004
Ferrocene	0.00020 ± 0.00002	0.030 ± 0.006	0.3 ± 0.1	0.023 ± 0.001	0.4 ± 0.1
1,1′-Dimethyl-ferrocene	0.00020 ± 0.00004	0.00030 ± 0.00002	0.00030 ± 0.00008	0.0038 ± 0.0009	0.07 ± 0.01
Ferrocene carboxaldehyde	0.10 ± 0.05	0.0040 ± 0.0004	0.5 ± 0.1	0.007 ± 0.001	0.03 ± 0.01
Ferrocene acetonitrile	0.020 ± 0.003	0.010 ± 0.002	0.07 ± 0.02	0.0014 ± 0.0001	0.14 ± 0.05

In general, it can be noted that mediators of the phenazine and phenathiosine series, which can penetrate into the cell [39], interact with microorganisms much faster than ferrocene derivatives. Further use of these mediators is advisable only when forming redox-active polymers based on them, since, in these systems, the heterogeneous transfer constant is inferior to the corresponding constants for ferrocene-series mediators. Among the ferrocene-series mediators immobilized in graphite powder, the mediator ferrocene is the most promising, since this system achieves the maximum rate constant of heterogeneous transfer to the electrode and, in most cases, a high-rate constant of interaction with the studied microorganisms (bacteria *P. yeei* and yeast *S. cerevisiae*).

**Figure 4:**
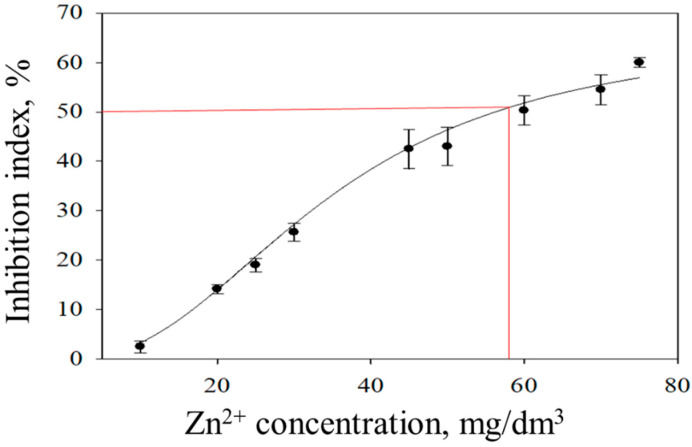
Inhibition curve for a bioelectrode based on the mediator ferrocene and bacteria *P. yeei*. Red line is IC_50_ value for the Zn^2+^ toxicant.

The choice of biomaterial for the formation of bioreceptor elements of a mediator biosensor was based on the sensitivity of microorganisms to harmful environmental factors. These include solutions of copper (II), zinc (II), lead (II), and cadmium (II) ions, as well as phenol and 4-nitrophenol, which are used as model toxicants for test objects in biotesting methods [15,16]. The sensitivity of the biosensor system to model toxicants was fixed by the index of inhibition of microorganisms. The inhibition index was taken to be the degree of reduction in the rate of glucose uptake by microorganisms in the presence and absence of an inhibitor. Inhibitory curves were constructed for each toxicant (Figure 4).

In the data obtained, we found concentrations of toxicants (IC_50_) that caused a 50% decrease in the activity of the biosensor receptor element. The inhibition index is given in Table 2. The results obtained were compared with similar models represented by microbial mediator biosensor systems [17,18,19,20,21,27], as well as biotesting methods based on various test objects, such as *Lemna* and *Vibrio fischeri* [15], and *Chlorella vulgaris* [16].

**Table 2:** Inhibitor solution concentration inducing a decrease of the receptor element activity by 50%.

Mediator/Biomaterial	Test Function	IC_50_ of the Toxicant, mg/dm^3^						Ref
		Pb^2+^	Cd^2+^	Cu^2+^	Zn^2+^	Phenol	*p*-Nitrophenol	
Ferrocene/ *S. cerevisiae*	Microbial metabolic activity decrease	6.1	– *	2.7	17.7	1.8	– *	This work
Ferrocene/ *E. coli*		0.8	8.9	47.6	46.4	17.6	5.8	This work
Ferrocene/ *Ps. Veronii*		169.8	– *	286.3	– *	13.5	– *	This work
Ferrocene/ *P. yeei*, *E. coli* association		7.3	6.6	23.8	2.3	8.1	29.2	This work
Ferrocene/ *P. yeei*		9.9	18.2	21.1	47.5	9.9	2.1	[27]
*p*-Benzoquinone/ *E. coli, B. subtilis, S. cerevisiae*		– *	20.5	16.5	– *	– *	– *	[17]
Menadione and potassium hexacyanoferrate(III)/ *S. cerevisiae*		34.6	13.9	10.1	– *	44.5	– *	[18]
Thionine/ *E. coli*		34.4	36.2	20.2	53.2	– *	– *	[19]
*p*-Benzoquinone/ *Psychrobacter* sp. isolated from activated sludge		110	47.3	2.6	10.9	– *	– *	[20]
Potassium hexacyanoferrate(III)/ activated sludge		– *	13.4	19.8	1.19	– *	– *	[21]
Duckweed (*Lemna*)	Inhibition of duckweed growth rate	5.5	0.33	0.33	0.9	– *	– *	[15]
*Vibrio fischeri*	Decrease of microbial bioluminescence intensity	36.0	52.5	34.4	4.64	– *	– *	[15]
*Chlorella vulgaris*	Evolution of oxygen in the gas phase	0.476	0.301	– *	– *	– *	– *	[16]

* not determined.

It can be concluded that *Ps. veronii* bacteria are the least sensitive to the studied toxicants, which makes further experiments with them impractical. Comparing the ferrocene–*S. cerevisiae* biosensor system with the described analogs, it can be said that the result of the toxic effect of the Pb^2+^ ion correlates with the data in [15] (in this work, we use the duckweed as a test object), as well as the data given for Cu^2+^ ions and described in [20]. In the ferrocene–*P. yeei* system, the result of the toxic effect of the Cu^2+^ ion correlates with the data presented in [19,21]. These two studies showed that the developed systems are sensitive to phenol solution. It should be noted that the ferrocene–*E. coli* system is the most sensitive to the action of Pb^2+^ ions, thus surpassing other standard test systems in sensitivity. In general, biosensor methods of toxicity determination are characterized by a lower sensitivity to heavy metals than when using duckweed as a test object [15]. Nevertheless, the development of biosensor analyzers is topical, due to the non-reactivity of the method and the possibility of continuous monitoring of water quality, as well as the high expressiveness of the analysis, which enables preventing various kinds of pollution.

By the results of this study, it can be concluded that single *P. yeei* and *E. coli* microorganisms can be used to form bioreceptor elements due to their higher sensitivity to the most frequently occurring toxicants in surface waters. To increase sensitivity, we considered the possibility of forming an association based on these microorganisms. This approach can make it possible to create receptor systems sensitive both to heavy metals and to organic toxicants. The yeast *S. cerevisiae* has a high sensitivity to toxicants, but in an association, it can eventually be displaced by bacteria due to their greater resistance to changes in environmental conditions [17]. For this reason, the association based on the yeast and bacteria was not used in further work. It seemed more expedient to form an association based on *P. yeei* and *E. coli*, because both of these microorganisms have the same specific growth rates, as well as the same linear growth phase build-up time, which makes it possible to ensure high stability of the analysis and to avoid the displacement of one microorganism by another during the long-term operation of the bioreceptor system [40] (Table 3). 

The receptor element based on associations of *P. yeei* and *E. coli* bacteria is not inferior in sensitivity to receptor elements based on single microorganisms (Table 2); at the same time, the bacterial association has a high sensitivity to both organic and inorganic toxicants.

### 3.3. Toxic Effects of Model Solutions on Characteristics of Ferrocene–P. yeei, Ferrocene–E. coli, and Ferrocene–S. cerevisiae Biosensor Systems

In terms of toxicological monitoring, it is necessary to make certain that the biomaterial used is suitable for analysis. For this purpose, it is recommended to pre-evaluate the sensitivity of the biomaterial to the reference toxicants, which were copper (II) ions and phenol. The concentration of toxicants was selected based on the degree of their inhibition of microorganisms, so that, in the presence of a model toxicant, the metabolic activity of cells decreased by 20% (Table 4). The choice of a toxicant concentration that reduces the metabolic activity of cells by 20% is due to the fact that this concentration of the toxicant allows the visualization of the biosensor response, and also the reduction of the load on the receptor element in long-term measurements. The degree of reversibility of the interaction of biomaterials with toxicants was preliminarily determined. This assessment included a parameter estimating how many times the inhibition index differed from 20% after holding the electrode in a solution of model toxicants.

It can be concluded that the chosen toxicants act reversibly on receptor systems based on single microorganisms and associations; therefore, they can be used as reference toxicants for assessing the quality of the formed working electrodes.

**Figure 5:**
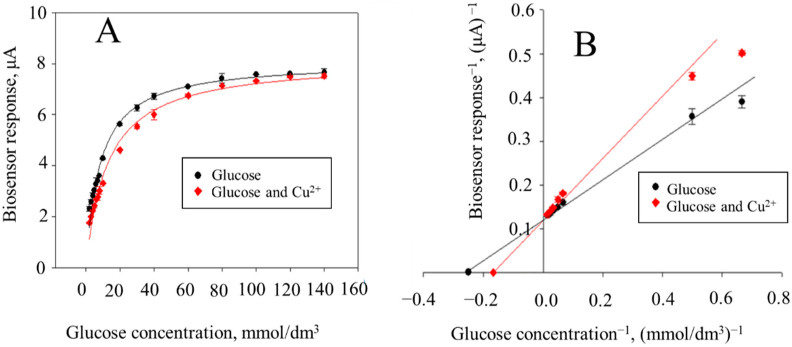
A form of the calibration curve (**A**) and the Lineweaver–Burk double reciprocal plot of the results obtained in the presence of Cu^2+^ (**B**).

In further experiments, we established the principle of the inhibitory effect of toxicants. For the ferrocene–*P. yeei* and ferrocene–*E. coli* biosensor systems, the Michaelis constants were determined (*K*_M_, the constant in the presence of substrate; *K*_MI_, the constant in the presence of substrate and inhibitor) and the maximum rate of the enzymatic reaction (*r*_max_, the maximum reaction rate without inhibitor; *r*_maxI_, the maximum reaction rate in the presence of inhibitor) proceeding from the calibration dependence parameters (Figure 5A). The type of inhibition was determined when processing the results obtained by the Lineweaver–Burk method (Figure 5B). The data are given in Table 5.

A competitive type of inhibition for copper (II) ion is observed in the *P. yeei*–ferrocene system. Despite the fact that this type of inhibition is typical for heavy metal ions, a similar phenomenon is also confirmed by studies presented in [41,42,43]. Copper ions in cell systems can act as catalysts for some redox reactions, enter the electron transfer chain, and form complex compounds [44]. In addition, in [43] it was shown that copper bound into a complex compound can compete for the electron–donor site of the enzyme NADH oxidoreductase, which is part of complex I of the electron transfer chain. Additionally, it was indicated in [43] that competitive inhibition includes an effect on an artificial acceptor, which in this work could be the mediator ferrocene. Furthermore, metals can form organic complex compounds structurally similar to substrates [45]. Considering the specifics of microorganisms, heavy metals can, as copper (II) ions, exhibit a competitive type of inhibition in relation to cell enzymes under conditions of the experiment. In the case of the *E. coli*–ferrocene system, the type of inhibition for Cu^2+^ ion proved to be mixed, which indicates that copper ions can bind both to the active site of the enzyme and have an effect outside the enzyme. Similar data on the type of inhibition were obtained in the study of the effect of Hg^2+^ on alcoholdehydrogenase [46]. A phenol solution exerts a mixed inhibition in bacteria-based biosensor systems. In [47], data were provided on the effect of cyclic compounds acting as an inhibitor of the enzyme phenylalanine ammonia lyase. The study revealed that phenol inhibits this enzyme in a mixed way. Identification of the inhibition mechanisms performed in this work will subsequently enable the choosing of a reversible model inhibitor for the receptor element based on an association. In this case, the effect of the toxicant will be different for the microorganisms used in the composition of the receptor element. The model inhibitor chosen in this way will allow for a consideration of possible inhibition mechanisms for the natural microflora when analyzing natural waters.

### 3.4. Characteristics of Developed Systems

**Figure 6:**
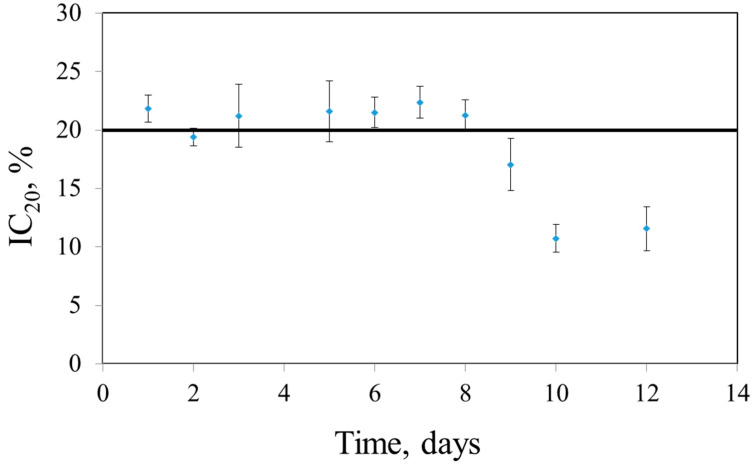
Long-time stability of the ferrocene–*P. yeei*–Cu^2+^ system.

When operating electrodes, it is important to evaluate reproducibility, as well as operational and long-term stability. Long-term stability characterizes the duration of biosensor operation over a certain period of time, which is ensured by the preservation of the vital activity of microorganisms on the surface of the electrode. Long-term stability was determined by measuring the degree of inhibition of microbial activity. For this, we daily measured the biosensor response at the concentration of the toxicant that caused a decrease in the respiratory activity of microorganisms by 20% (IC_20_). Between measurements, the electrode was kept in a phosphate buffer solution (pH 6.8). If the sensor response decreased by less than 20%, the electrode was considered unfit for work. The obtained dependences are shown in Figure 6. Despite the reversible short-term interaction between the receptor element and the model toxicant, the decrease in the receptor element activity on the 9th day is probably due to the cumulative toxic effect of Cu^2+^ ions. The results are given in Table 6.

The system based on the ferrocene mediator and *P. yeei* bacteria had the greatest stability in biosensor operation. The assessment of the operational stability of the biosensor was carried out by introducing a solution of a substrate of the same concentration and an inhibitor causing a 20% decrease in the respiratory activity (IC_20_). A quantitative characteristic of operational stability is the relative standard deviation in repeated measurements (15 times) of the standard sample (95% confidence probability). The results obtained are presented in Table 6. From these data, it follows that the most stable bioreceptor element for long–term measurements is the ferrocene–*P. yeei*–Cu^2+^ system.

The reproducibility of the signals of the receptor elements was evaluated by the magnitude of the relative standard deviation of responses from a series consisting of seven electrodes at the introduction of a model solution (95% confidence probability). Based on the results obtained, the best reproducibility indicator can be said to be the ferrocene–*P. yeei*–Cu^2+^ system.

### 3.5. Determination of Integral Toxicity of Wastewater Samples

Three wastewater samples were analyzed using the developed mediator biosensors based on microorganisms and the method of biotesting (the test object was *Chlorella*, the reference method). Three samples of river water were used. The registered parameter was the toxicity index defined as the degree of a decrease in the activity of the biosensor bioreceptor element and the degree of inhibition of chlorella growth. The results obtained are presented in Table 7. The criterion for the toxicity of a water sample is a decrease in the average optical density compared to the control by 20% or more, or an increase in the average optical density by 30% or more. The detectable parameter was the toxicity dilution factor (*TDF*).

The *TDF* values calculated by Formulas (3) and (4) are given in Table 7.

According to the data obtained, it can be concluded that Sample 1 is highly toxic. The best correlation with the results of the standard method in the analysis of natural waters was achieved using ferrocene–*E.coli* and ferrocene–association *P. yeei*/*E.coli* biosensor systems. The ferrocene–*P. yeei* biosensor system is not suitable for *TDF* determination due to its rather low sensitivity to toxicants that can occur in analyzed samples. At the same time, the ferrocene–*S. cerevisiae* biosensor system possesses a high sensitivity to toxicants of the sample, due to which the results do not correlate with the data of the standard method. The results obtained in the experiment suggest that the ferrocene–*E. coli* and ferrocene– association *P. yeei*/*E. coli* biosensor systems can be successfully used for determining the general toxicity of aqueous media. The developed bioanalytical systems can serve as prototypes for commercial rapid-assay toxicity analyzers. The main limitation of wide application of the developed receptor elements is long-time stability: 3 days for *E. coli*/ferrocene system and 4 days for *P. yeei*, *E. coli*/ferrocene.

## 4. Conclusions

This study established that the analytical signal of the biosensor in a rapid assay could be adequately treated as a degree of inhibition of the receptor-element respiratory activity as the result of a glucose oxidation rate decrease in the presence of an inhibitor. This enabled an accounting for the effects of substances that could occur in the sample and be oxidized by the biomaterials of the receptor system. The values of EC_50_ were found for four heavy metals (Cu^2+^, Zn^2+^, Pb^2+^, Cd^2+^) and two phenols (phenol and p-nitrophenol). The bacteria *E. coli* and *P. yeei* were the most sensitive to the action of the model toxicants: Cu^2+^, Pb^2+^, and phenol. Therefore, the use of these microorganisms as biomaterials in the development of an amperometric biosensor for integral toxicity determination is more preferable. As the growth parameters of these microorganisms are close, a stable association formed on their basis can be assumed. 

The developed ferrocene–*E. coli* and ferrocene–*P. yeei*/*E. coli* systems have high sensitivities to solutions of heavy metals and organic compounds, as compared with other analogs presented in other publications. Mediator biosensors based on an association of *E. coli* and *P. yeei* bacteria for integral toxicity assays are reagent-free. The method enables much faster results than other biotesting methods; the assay results are consistent with those of the standard biotesting using *Chlorella vulgaris* algae as a test object. 

The developed biosensor allows for the evaluation of the impact of polluted natural water to the ecosystem via assessment of impacts on bacteria and yeasts in the receptor system. These systems can be used in toxicological monitoring of natural waters.

## Figures and Tables

**Table 3:** Parameters of microbial growth curves.

Growth Phase	*P. yeei* VKM B-3302	*E. coli* K802	*S. cerevisiae* VKM Y-1173	*Ps. veronii* DSM 11331T
Lag phase, h	0–2	0–2	0–2	0–6
Exponential phase, h	2–4	2–4	2–4	6–12
Linear growth phase, h	4–16	4–10	4–8	12–20
Transition phase (growth slowdown phase), h	16–24	10–20	8–17	20–36
Stationary phase, h	24–30	20–30	17–30	36–52
Specific growth rate, h^−1^	0.6 ± 0.2	0.6 ± 0.1	0.71 ± 0.04	0.265

**Table 4:** Concentrations of toxicant solutions at which the metabolic activity of the bioreceptor element decreases by 20%.

Biomaterial/Mediator	Toxicant Solution	Toxicant Concentration at which the Metabolic Activity of Cells Decreases by 20%, IC20, mg/dm^3^	Reversibility Degree, %
*P. yeei*–ferrocene	Cu^2+^	10	96 ± 4
	Phenol	4	96 ± 2
*E. coli*–ferrocene	Cu^2+^	24	97 ± 2
	Phenol	8	94 ± 4
*S. cerevisiae*–ferrocene	Cu^2+^	1.5	94 ± 3
	Phenol	1	93 ± 5
*P. yeei/E.coli* association–ferrocene	Zn^2+^	2.3	96 ± 2
	Phenol	8.1	93 ± 4

**Table 5:** Michaelis–Menten equation parameters in the presence of reference toxicants for *P. yeei*–ferrocene and *E. coli*–ferrocene biosensor systems.

Biomaterial/ Mediator	Toxicant	*K*_M_, mmol/dm^3^	*K*_MI_ mmol/dm^3^	*r*_max_, μA	*r*_maxI_, μA	Inhibition Type
*P. yeei*/ ferrocene	Cu^2+^	8 ± 2	13 ± 2	8.1 ± 0.4	8.1 ± 0.3	Competitive
	Phenol	9 ± 2	6 ± 1	5.3 ± 0.3	4.5 ± 0.3	Mixed
*E. coli*/ ferrocene	Cu^2+^	2.1 ± 0.9	0.5 ± 0.05	2.5 ± 0.3	1.97 ± 0.03	Mixed
	Phenol	2.1 ± 0.5	1.0 ± 0.3	2.9 ± 0.2	2.3 ± 0.2	Mixed
*S. cerevisiae*/ ferrocene	Cu^2+^	1.9 ± 0.1	1.6 ± 0.2	1.3 ± 0.1	0.77 ± 0.04	Mixed
	Phenol	3.2 ± 0.9	5 ± 2	1.0 ± 0.2	1.1 ± 0.4	Competitive

**Table 6:** Analytical characteristics of the developed systems.

Biomaterial/Mediator	Toxicant	Long-Time Stability, Days	Operational Stability, %	Reproducibility, %
*P. yeei*/ferrocene	Cu^2+^	9	4.9	4.5
	Phenol	10	5.3	4.8
*E. coli*/ferrocene	Cu^2+^	3	6.92	7
	Phenol	3	10.81	9
*S. cerevisiae*/ferrocene	Cu^2+^	5	6.9	4.9
	Phenol	5	11.5	5.1
*P. yeei, E. coli*/ferrocene	Cu^2+^	4	7.3	5.2
	Phenol	4	10.3	9.6

**Table 7:** The results of calculating *TDF* for analyzed samples.

Method of Analysis	*TDF* of Analyzed Samples		
	Sample No 1	Sample No 2	Sample No 3
Chlorella vulgaris biotesting	56-fold (highly toxic)	21-fold (toxic)	7-fold (mid toxic)
Ferrocene–*E. coli* biosensor	63-fold (highly toxic)	16-fold (toxic)	5-fold (mid toxic)
Ferrocene–*P. yeei* biosensor	25-fold (toxic)	6-fold (mid toxic)	2-fold (low toxic)
Ferrocene–*S. cerevisiae* biosensor	45-fold (highly toxic)	60-fold (highly toxic)	32-fold (highly toxic)
Ferrocene–*E. coli/P. yeei* biosensor	60-fold (highly toxic)	19-fold (toxic)	8-fold (toxic)

## Data Availability

Not applicable.
